# Circulating metabolites and molecular lipid species are associated with future cardiovascular morbidity and mortality in type 1 diabetes

**DOI:** 10.1186/s12933-022-01568-8

**Published:** 2022-07-18

**Authors:** Luis F. Ferreira-Divino, Tommi Suvitaival, Viktor Rotbain Curovic, Nete Tofte, Kajetan Trošt, Ismo M. Mattila, Simone Theilade, Signe A. Winther, Tine W. Hansen, Marie Frimodt-Møller, Cristina Legido-Quigley, Peter Rossing

**Affiliations:** 1 Steno Diabetes Center Copenhagen, Borgmester Ib Juuls Vej 83, 2730 Herlev, Denmark; 2 University of Copenhagen, Copenhagen, Denmark; 3 The Department of Medicine, Herlev-Gentofte Hospital, Copenhagen, Denmark

**Keywords:** Type 1 diabetes, Cardiovascular disease, Cardiovascular mortality, Lipidomics, Metabolomics, Omics

## Abstract

**Background:**

Cardiovascular disease remains the leading cause of mortality in individuals with diabetes and improved understanding of its pathophysiology is needed. We investigated the association of a large panel of metabolites and molecular lipid species with future cardiovascular events in type 1 diabetes.

**Methods:**

The study included 669 individuals with type 1 diabetes. Non-targeted serum metabolomics and lipidomics analyses were performed using mass spectrometry. Data on cardiovascular events (cardiovascular mortality, coronary artery disease, stroke, and peripheral arterial interventions) were obtained from Danish Health registries and analyzed by Cox hazards models. Metabolites and molecular lipid species were analyzed in univariate models adjusted for false discovery rate (FDR). Metabolites and molecular lipid species fulfilling a p_FDR_ < 0.05 were subsequently analyzed in adjusted models including age, sex, hemoglobin A_1c_, mean arterial pressure, smoking, body mass index, low-density lipoprotein cholesterol, estimated glomerular filtration rate, urinary albumin excretion rate and previous cardiovascular disease. Analyses of molecular lipid species were further adjusted for triglycerides and statin use.

**Results:**

Of the included participants, 55% were male and mean age was 55 ± 13 years. Higher 4-hydroxyphenylacetic acid (HR 1.35, CI [1.01–1.80], p = 0.04) and lower threonine (HR 0.81, CI [0.67–0.98] p = 0.03) were associated with development of cardiovascular events (n = 95). In lipidomics analysis, higher levels of three different species, diacyl-phosphatidylcholines (PC)(36:2) (HR 0.82, CI [0.70–0.98], p = 0.02), alkyl-acyl-phosphatidylcholines (PC-O)(34:2) (HR 0.76, CI [0.59–0.98], p = 0.03) and (PC-O)(34:3) (HR 0.75, CI [0.58–0.97], p = 0.03), correlated with lower risk of cardiovascular events, whereas higher sphingomyelin (SM)(34:1) (HR 1.32, CI [1.04–1.68], p = 0.02), was associated with an increased risk.

**Conclusions:**

Circulating metabolites and molecular lipid species were associated with future cardiovascular events in type 1 diabetes. While the causal effect of these biomolecules on the cardiovascular system remains unknown, our findings support that omics-based technologies, although still in an early phase, may have the potential to unravel new pathways and biomarkers in the field of cardiovascular disease in type 1 diabetes.

**Supplementary Information:**

The online version contains supplementary material available at 10.1186/s12933-022-01568-8.

## Background

Despite better control of established risk factors, there remains a substantial excess risk of cardiovascular (CV) morbidity and mortality in individuals with type 1 diabetes [1, 2]. Thus, a better understanding of the underlying pathophysiology to improve risk assessment and intervention in individuals with type 1 diabetes is needed. Omics-based technologies may allow for further understanding of the pathophysiology underlying cardiovascular disease (CVD) in type 1 diabetes and can potentially lead to identification of novel biomarkers and targets for new therapies or implementation of existing therapies in high risk individuals.

Excess CV mortality and reduced lifespan is mainly driven by an earlier onset and more aggressive progression of atherosclerosis in type 1 diabetes compared to the general population [3, 4]. Atherosclerosis is a process triggered by inflammation in the endothelium causing binding of macrophages that take up oxidized low-density lipoproteins and transform to foam cells. Foam cells together with T cells and calcified smooth muscle cells cluster into atherosclerotic plaques [5]. As circulating biomolecules are in direct contact with the endothelium and may be involved in the atherosclerotic process, the study of metabolic pathways using novel, mass-spectrometry-based omics methods is of special interest in the field of CVD. These technologies allow for a more comprehensive description of the metabolic pathways compared with traditional biomarkers. Omics methods, despite early in its technical and scientific evolution, have already demonstrated substantial potential for a deepened understanding in CVD [6]. Furthermore, we have previously provided new insights into how metabolites and molecular lipid species are associated with microvascular complications in type 1 diabetes [7, 8].

In the present study, we investigated a large panel of serum metabolites and molecular lipid species in association with the risk of atherosclerotic CV morbidity and mortality in individuals with type 1 diabetes.

## Methods

### Study population

In total, 676 individuals with type 1 diabetes followed at the outpatient clinic at Steno Diabetes Center Copenhagen were included in a cross-sectional study in 2009–2011 with planned follow up for kidney and cardiovascular events (CVE). The details of the original study have previously been published [9]. The cohort was selected to include individuals with a wide range of albuminuria; hence, 316 individuals with normoalbuminuria, 169 with microalbuminuria and 191 with macroalbuminuria were included. In 2017, we performed metabolomics and lipidomic analyses on baseline serum samples from 637 (94%) and 669 (99%) of the participants, respectively. The study was performed in accordance with the Declaration of Helsinki and approved by the local ethics committee in the Capital Region of Denmark. All participants gave written informed consent to the original study and the research biobank.

### Baseline measures

Hemoglobin A_1c_ (HbA_1c_), low-density lipoprotein (LDL) cholesterol, total plasma triglycerides and serum creatinine levels were measured in the laboratory at Steno Diabetes Center Copenhagen using standardized methods. Urinary albumin excretion rate (UAER) was measured in three consecutive 24-h urine collections. The estimated glomerular filtration rate (eGFR) was calculated from serum creatinine using the Chronic Kidney Disease Epidemiology Collaboration (CKD-EPI) equation [10]. Brachial blood pressure was measured in the sitting position after at least 10 min rest with an automatic validated oscillometric device and an appropriately sized cuff. Body mass index (BMI) was measured during the study visit. Current users of > 1 cigarettes, cigars or pipes per day were classified as smokers and all others as non-smokers. Information on medication and previous CVD was collected from questionnaires and cross-checked in electronic medical records by the investigator.

### Omics analyses

The serum samples were immediately stored at − 80 °C until analysis in 2017. The methods used for analyzing metabolomics and lipidomics have been described in previous papers [8, 11]. In brief, a non-targeted two-dimensional gas chromatography time-of-flight mass-spectrometry method Leco Pegasus 4D GC × GC-TOFMS instrument (Leco Corp., MI, USA) was used for the metabolomics analysis which subsequently identified 75 metabolites included in the data analysis. The lipidomics analysis was performed with an ultra-high-performance liquid chromatography quadrupole time-of-flight mass spectrometry method UHPLC-QTOF/MS (Agilent Technologies, Santa Clara, CA, USA) [12, 13] identifying 106 named molecular lipid species. Metabolites or molecular lipid species with equal to or less than 20% missing or undetectable values were included in the analysis and all missing values were imputed with the k-nearest neighbor algorithm. Finally, all values were log2-transformed [14].

### Definition of CVE during follow-up

ICD-10 diagnoses (www.who.int/classifications/icd/en/), procedural codes (according to the Nordic Classification of Surgical Procedures (NCSP): https://medinfo.dk/sks/brows.php) and date of death were obtained from the Danish National Health Register until December 31, 2016 [15, 16]. Data regarding cause of death was available from the Danish National Death Register until December 31, 2015. Five endpoints were included: (1) any CVE including all of the endpoints introduced next [2–5], (2) CV mortality, (3) coronary artery disease including non-fatal myocardial infarction and coronary revascularization (percutaneous arterial intervention or coronary bypass grafting), (4) non-fatal stroke, and (5) peripheral arterial interventions including amputations. All included ICD-10 codes and NCSP procedural codes are presented in full in Additional file 1: Table S1. For the analyses of the any CVE, only the first event was included, even if participants experienced consecutive endpoints. Except when an unambiguous non-CV cause of death was reported, all deaths were defined as CV mortality, an approach that has formerly been applied [17].

### Statistical analyses

Continuous variables were reported as mean ± standard deviation (SD) if normal distributed and as median [interquartile range (IQR)] if non-normal distributed. The non-normal distributed variables were log2-transformed before analyses. Categorical variables were presented as total numbers with corresponding percentages. To test for differences in baseline characteristics in the population according to previous CVD, un-paired t-tests were applied for continuous variables and the Chi-Squared tests for categorical variables. A two-tailed *p* value of < 0.05 was considered statistically significant. Baseline data were analyzed in SAS Enterprise Guide version 7.15 and all other data were analyzed with R-3.4.2.

Associations of the metabolites and molecular lipid species with all CV endpoints were examined longitudinally with the Cox proportional hazards model using the R-package survival. Hazard ratios (HRs) with 95% confidence interval (CI) per 1 SD increase on the log2-scale were computed for all endpoints. Prioritization of the compounds was done using a stepwise selection in which all measured metabolites (Additional file 1: Table S2) and molecular lipid species (Additional file 1: Table S3) were tested with the crude model and p-values were corrected for false discovery rate (FDR) with the Benjamini–Hochberg method. Thereafter, metabolites and molecular lipid species fulfilling a p_FDR_ < 0.05 in univariate analyses were selected for analysis in adjusted models including sex, baseline age, HbA_1c_, mean arterial pressure, smoking, BMI, LDL cholesterol, eGFR, UAER and previous CVD. In addition, total p-triglycerides and statin use were included in analyses with molecular lipid species. HRs of the metabolites or molecular lipid species were visualized in forest plots grouped by endpoint using R-package ggplot2.

## Results

### Baseline characteristics

The 669 participants had a mean age of 55 ± 13 years, a median [IQR] diabetes duration of 35 [24–44] years, 55% (n = 370) were men and 21% (n = 143) had previous CVD at baseline (Table 1). Individuals with previous CVD were older, had a longer diabetes duration, higher blood pressure, higher HbA_1c_, lower LDL cholesterol, higher triglycerides, lower eGFR and higher UAER. Moreover, individuals with previous CVD were more frequently prescribed antihypertensive agents, statin treatment, and aspirin or clopidogrel (p < 0.01). During follow-up, 95 individuals had at least one event CVE endpoint (any CVE), 11 died from CV causes, 45 were diagnosed with coronary artery disease, 23 experienced a non-fatal stroke, and 35 had a peripheral arterial intervention performed. Median follow-up for the any CVE endpoint was 5.3 [4.8–5.7] years.

**Table 1:** Baseline characteristics overall and stratified by previous CVD

	All *(n* = *669)*	Previous CVD *(n* = *143)*	No previous CVD *(n* = *526)*	P-value
Female, n (%)	299 (45)	60 (42)	239 (45)	0.46
Age, years	55 ± 13	61 ± 9	53 ± 13	< 0.01
Diabetes duration, years	35 [24–44]	42 [34–49]	33 [19–42]	< 0.01
BMI, kg/m^2^	26 ± 6	25 ± 4	25 ± 6	0.97
Systolic blood pressure (mmHg)	132 ± 18	135 ± 20	131 ± 17	0.01
Diastolic blood pressure (mmHg)	74 ± 9	71 ± 9	75 ± 9	< 0.01
Mean arterial pressure (mmHg)	93 ± 10	92 ± 11	94 ± 10	0.15
HbA_1c_, % (mmol/mol)	8.0 ± 1.2 (64 ± 13)	8.2 ± 1.2 (67 ± 13)	8.0 ± 1.2 (64 ± 13)	0.02
Total cholesterol, mmol/l	4.7 ± 0.9	4.6 ± 0.9	4.7 ± 0.9	0.47
LDL cholesterol, mmol/l	2.5 ± 0.8	2.3 ± 0.7	2.5 ± 0.8	0.01
HDL cholesterol, mmol/l	1.7 ± 0.5	1.7 ± 0.5	1.7 ± 0.5	0.93
Triglycerides, mmol/l	1.0 [0.7–1.3]	1.0 [0.8–1.6]	0.9 [0.7–1.3]	< 0.01
eGFR, ml/min/1.73 m^2^	81 ± 26	67 ± 25	85 ± 24	< 0.01
UAER, mg/24-h	18 [8–64]	34 [11–124]	15 [8–52]	< 0.01
Smokers, n (%)	139 (21)	27 (19)	112 (21)	0.53
Antihypertensives, n (%)	481 (72)	135 (94)	346 (66)	< 0.01
Statins, n, %	401 (60)	120 (84)	281 (54)	< 0.01
Aspirin or Clopidogrel n (%)	352 (53)	125 (88)	227 (64)	< 0.01
*Follow-up*				
Any CVE*, n (%)	95 (14)	55 (38)	40 (8)	< 0.01
CV mortality, n (%)	11 (2)	8 (6)	3 (1)	< 0.01
Coronary artery disease^†^, n (%)	45 (7)	27 (19)	18 (3)	< 0.01
Non-fatal stroke, n (%)	23 (3)	12 (8)	11 (2)	< 0.01
Peripheral arterial interventions, n (%)	35 (5)	21 (15)	14 (3)	< 0.01

Data are n (%, rounded), mean ± SD or median [IQR]. HbA_1c_ hemoglobin A_1c_, eGFR estimated glomerular filtration rate, UAER urinary albumin excretion rate. *Any CVE consisted of CV mortality, non-fatal stroke, coronary artery disease and peripheral arterial interventions. ^†^Coronary artery disease includes non-fatal myocardial infarction and coronary revascularization. P for difference between participants with or without previous CVD were calculated using un-paired t-test for continuous variables and the χ2-test for categorical variables

### CV risk in relation to metabolites

**Fig. 1:**
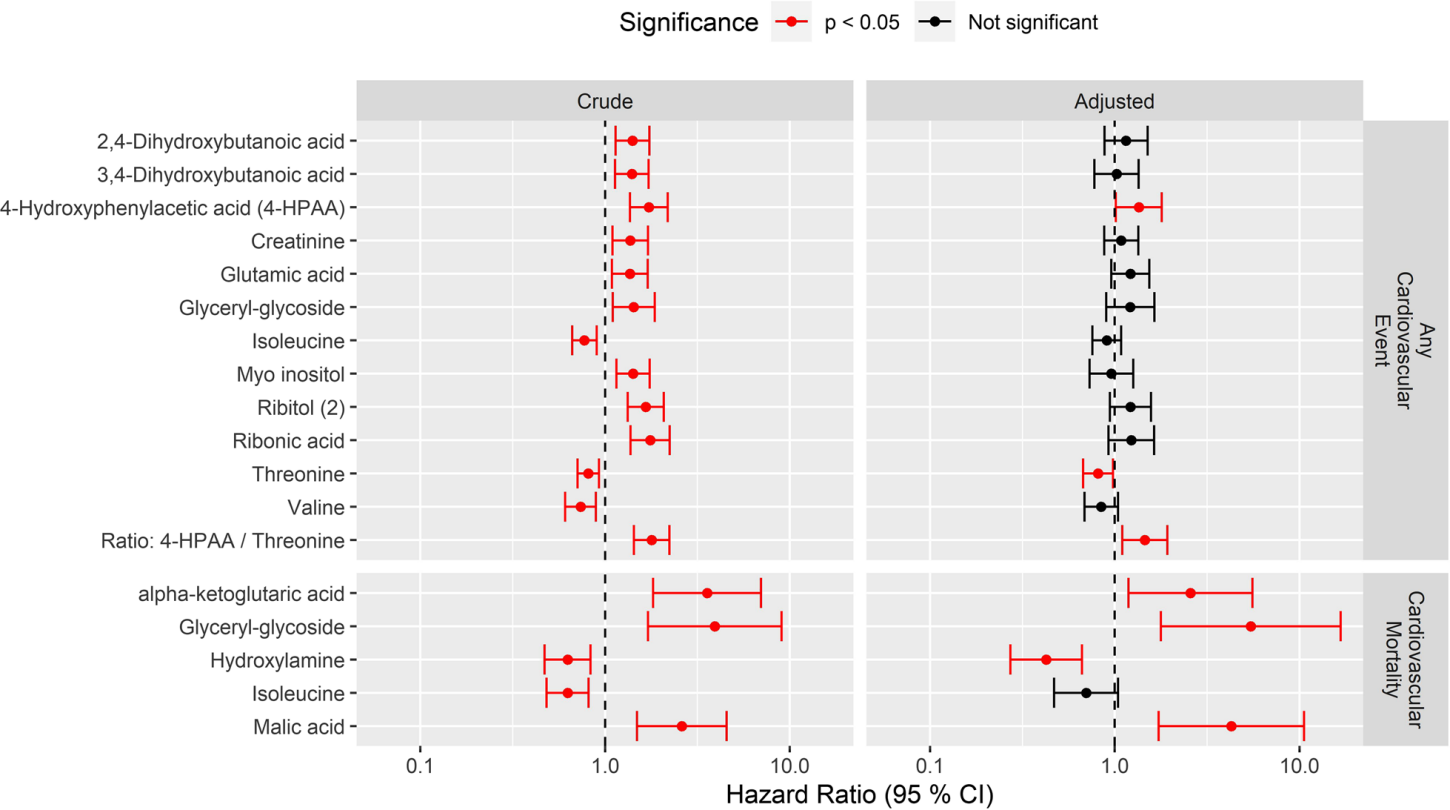
Association between metabolite level at baseline and cardiovascular events during the follow-up. Shown are the hazard ratio (x-axis) per 1 SD of the metabolite level) of metabolites (rows) associated with any cardiovascular event (upper panel) and cardiovascular mortality (lower panel). Association and 95% confidence intervals are shown from the crude model (left panel) and from the model adjusted for clinical covariates; sex, baseline age, Hemoglobin A_1c_, mean arterial pressure, smoking, BMI, LDL cholesterol, estimated glomerular filtration rate, urinary albumin excretion rate and previous cardiovascular disease (right panel). Metabolites with a crude association at FDR < 5% are included in the figure, and associations with p < 0.05 are shown in red

**Fig. 2:**
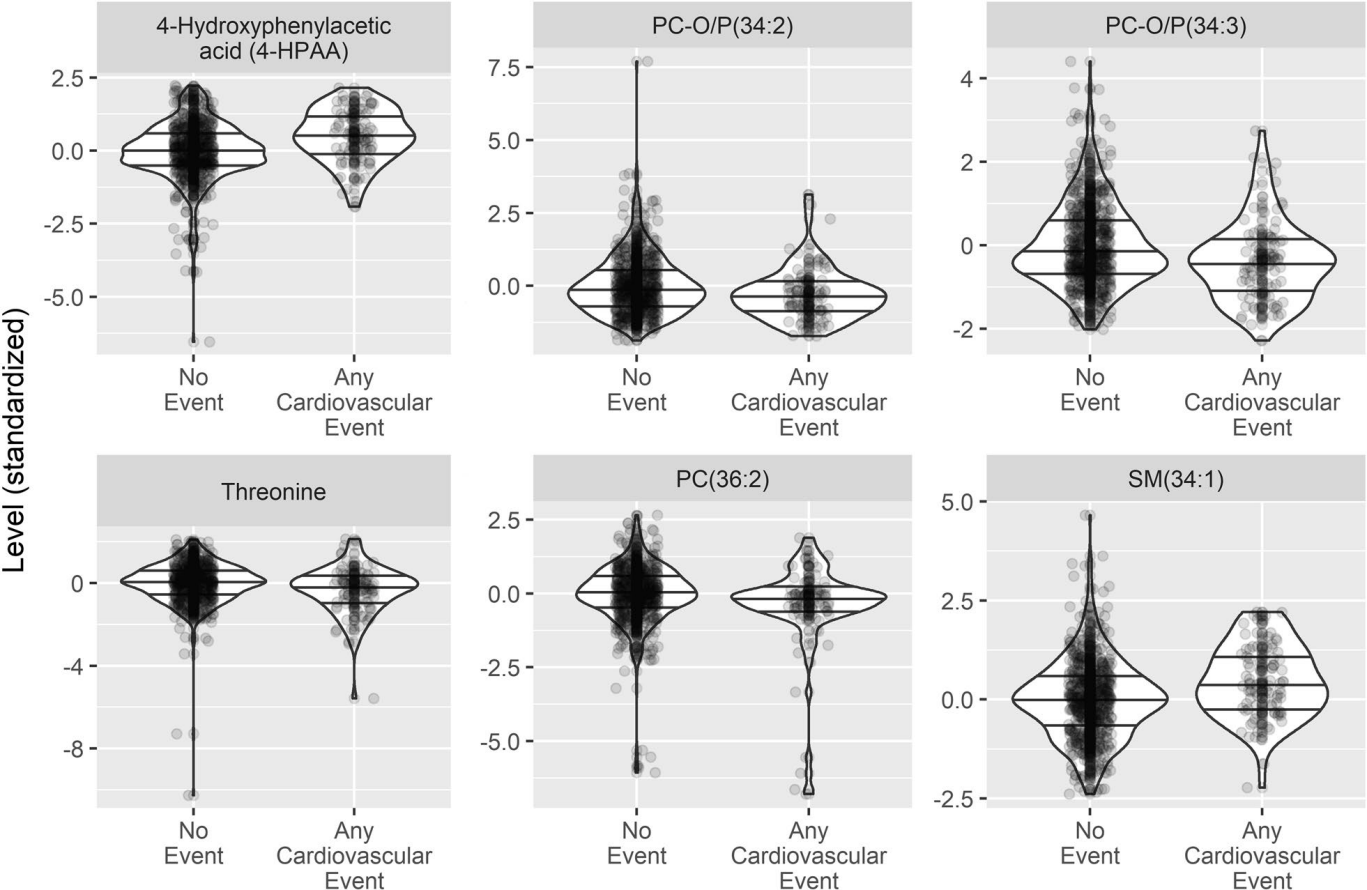
Baseline blood levels (y-axis) of CVD-associated molecules, stratified by participants experiencing any cardiovascular event (n = 95) or not. Plasma metabolite or molecular lipid species level in an individual is shown with a dot, and the population distribution is shown with a violin plot, where horizontal lines indicate the quartiles. Metabolites are shown in the first column and molecular lipid species in the two other columns

A total of 75 metabolites were included in the analyses. After correction for multiple testing, twelve metabolites were identified as significantly associated with any CVE in the crude analysis. In models adjusted for clinical covariates, higher 4-hydroxyphenylacetic acid (4-HPAA) (HR 1.35, CI [1.01–1.80], p = 0.04) and lower threonine (HR 0.81, CI [0.67–0.98] p = 0.03) were significantly associated with any CVE endpoint. The metabolite ratio of 4-HPAA and threonine was also significantly associated with any CVE endpoint (adjusted HR 1.45, CI [1.10–1.92], p < 0.01) (Fig. 1, upper panel). Baseline levels of the two metabolites associated with any CVE are shown in Fig. 2 (first column), when stratified by individuals having experienced any CVE or not.

Five metabolites were associated with CV mortality in crude models after correction for multiple testing (Fig. 1, lower panel). In multivariable analyses, four of these metabolites remained associated with CV mortality; higher alpha-ketoglutaric acid (α-KG) (HR 2.75, CI [1.19–5.57], p = 0.02), glyceryl-glycoside (HR 5.44, CI [1.77–16.7], p < 0.01) and malic acid (HR 4.27, CI [1.72–10.6], p < 0.01) were associated with an increased risk of CV mortality. On the contrary, higher hydroxylamine (HR 0.43, CI [0.27–0.66], p < 0.01) was significantly associated with a lower risk of CV mortality. In the analyses of other specific endpoints, no metabolites were significantly associated with the risk of coronary artery disease, non-fatal stroke or peripheral arterial intervention.

### CV risk in relation to molecular lipid species

**Fig. 3:**
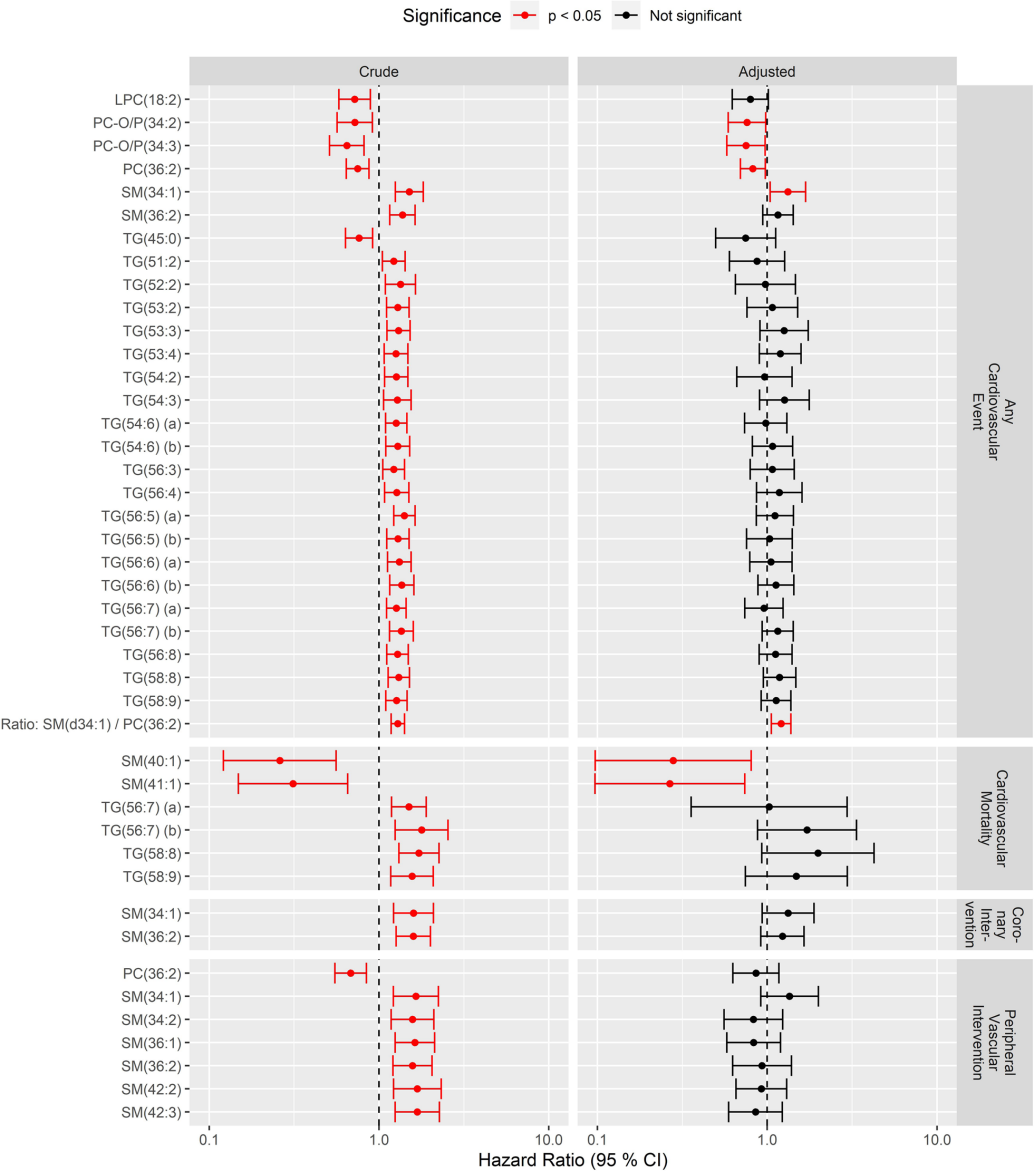
Association between lipid level at baseline and cardiovascular events during follow-up. Shown are hazard ratio (x-axis) per 1 SD of the lipid level) of molecular lipid species (rows) associated with any cardiovascular event (upper panel) and cardiovascular mortality (lower panel). Association and 95% confidence intervals are shown from the crude model (left panel) and from the model adjusted for clinical covariates: sex, baseline age, Hemoglobin A_1c_, mean arterial pressure, smoking, BMI, LDL cholesterol, total plasma triglycerides, estimated glomerular filtration rate, urinary albumin excretion rate, previous cardiovascular disease, and statin use (right panel). Molecular lipid species with a crude association at FDR < 5% are included in the figure, and associations with p < 0.05 are shown in red

In the lipidomic analysis, a total of 106 known molecular lipid species were identified belonging to five major classes: diacyl-phosphatidylcholines (PCs), lyso-phosphatidylcholines (LPCs) alkyl-acyl-phosphatidylcholines (PC-Os), sphingomyelins (SMs) and triacylglycerols (TGs). In crude models, one PC, one LPC, two PC-Os, two SMs and twenty-one TGs were associated with risk of any CVE, after correction for multiple testing. Four of these molecular lipid species, PC(36:2) (HR 0.82, CI [0.70–0.98], p = 0.02), PC(O-34:2) (HR 0.76, CI [0.59–0.98], p = 0.03), PC(O-34:3) (HR 0.75, CI [0.58–0.97], p = 0.03) and SM(d34:1) (HR 1.32, CI [1.04–1.68], p = 0.02), remained associated with any CVE endpoint after adjustment for clinical covariates. The molecular lipid species ratio of the two lipids with strongest association, SM(d34:1) and PC(36:2), was significantly associated with the any CVE endpoint (adjusted HR 1.21, CI [1.06–1.38], p < 0.01) (Fig. 3, upper panel). Baseline levels of the molecular lipid species associated with any CVE are shown in Fig. 2 (two last columns), when stratified by individuals having experienced any CVE or not.

Six molecular lipid species: two SMs and four TGs, were associated with CV mortality in crude models; however, only the associations for the sphingomyelins—SM(d40:1) (HR 0.28, CI [0.10–0.80], p = 0.02) and SM(d41:1) (HR 0.27, CI [0.10–0.74], p = 0.01) remained in the adjusted model (Fig. 3, second panel from the top). Higher SM(d34:1) and SM(d36:2) were associated with coronary artery disease in crude models; however, associations were lost after adjustment for clinical covariates (Fig. 3, third panel from the top). No molecular lipid species were associated with the risk of non-fatal stroke. Higher SM(d34:1) and SM(d36:2) as well as four other SMs and one PC were associated with peripheral vascular interventions in crude models (Fig. 3, bottom panel). However, these did not remain significant after adjustment for clinical covariates.

### Correlations between circulating metabolites, molecular lipid species and clinical lipid measurements

We investigated the cross-sectional correlations between circulating metabolites and lipid species, with LDL, HDL, TG, total cholesterol and statin treatment. Only weak correlation between different circulating metabolites and LDL, HDL, TG and total cholesterol were seen (Additional file 1: Fig. S1). However, we demonstrated positive correlations between PCs and SMs and total cholesterol and negative correlations between TGs and HDL (Additional file 1: Fig. S2). In general, no correlations between circulating metabolites or molecular lipid species with lipid lowering therapy were shown (Additional file 1: Figs. S1 and S2).

## Discussion

We investigated the longitudinal associations between serum metabolites, molecular lipid species and the risk of CV morbidity and mortality in a large type 1 diabetes cohort. The cohort is characterized by long diabetes duration, high prevalence of previous CVD and a wide range of albuminuria. The main findings were that (1) two metabolites and four molecular lipid species were associated with any CVE after adjustment for relevant clinical covariates, and (2) four metabolites and two molecular lipid species were associated with CV mortality after adjustment.

In the present study, higher 4-HPAA was associated with any future CVE. As we are aware, the association between 4-HPAA and risk of CVD in individuals with type 1 diabetes has not previously been examined. Circulating 4-HPAA is an active metabolite of the amino acid metabolism as phenylalanine is converted to tyrosine [18], and further converted into 4-HPAA or nitrotyrosine [19, 20]. While little is known about 4-HPAA as a risk factor for CVD, nitrotyrosine has been proposed as an inflammatory marker for development of atherosclerosis and subsequent coronary artery disease [21]. Additionally, we identified higher threonine, an essential amino acid, to be associated with a lower risk of any CVE. In a population based cross-sectional study, higher threonine level was associated with a better lipid profile, supplementing our longitudinal findings [22].

Higher levels of two metabolites of the mitochondrial energy metabolism, α-KG and malic acid, both intermediates of the tricarboxylic acid (TCA) cycle, were associated with an increased risk of CV mortality in our study. Alterations in the mitochondrial energy metabolism have been shown to relate to the development of diabetic cardiomyopathy, a common complication in type 1 diabetes [23–25]. The association between α-KG, malic acid and CV mortality has not previously been assessed longitudinally in type 1 diabetes, however, in earlier studies the former has been associated with increased risk of re-hospitalization and mortality in acute heart failure patients [26], and the latter with increased risk of heart failure [27]. Dunn, et al., hypothesized that alterations in mitochondrial energy metabolism could lead to a decreased flux of intermediate metabolites through the TCA cycle, subsequently leading to an overflow of these metabolites into the circulation [28]. Moreover, we demonstrated a novel finding between higher glyceryl-glycoside and CV mortality. Higher hydroxylamine, an inorganic vasorelaxant [29], was associated with a lower risk of CV mortality in our study. Whereas the role of hydroxylamine in the pathophysiology of CVD remains unknown, it could be hypothesized that the vasorelaxant function mediates antihypertensive properties in the CV system. While the present study is not designed to answer any of the above-mentioned hypotheses, the findings warrant further studies to better understand the underlying mechanisms.

Regarding the analyses of the molecular lipid species, we demonstrated that higher levels of three different molecular lipid species, PC(36:2), PC (O-34:2) and PC (O-34:3), correlated with lower risk of any CVE, whereas higher SM(34:1) was associated with an increased risk. We have previously shown that altered lipidome profiles were associated with microvascular complications in the same cohort. Particularly, Tofte, et al., found the same PC-Os (PC(O-34:2) and PC(O-34:3)) were associated with lower risk of event in a combined renal endpoint (≥ 30% decrease in eGFR, end stage kidney disease and all-cause mortality). Moreover, higher SM(d40:1) and SM(d41:1), two SMs associated with lower risk of CV mortality in the present study, were at baseline lower in individuals with macroalbuminuria compared to normoalbuminuria and associated with a lower risk of a combined renal endpoint; indicating a potential overlap in the molecular pathophysiology associated with micro- and macrovascular disease, independent of traditional risk factors. However, no differences in circulating metabolites or lipid species were demonstrated in individuals with microalbuminuria compared to normal albuminuria, or with UAER analyzed as a continuous variable at baseline [7, 11]. Similarly, in a recent study including a small cohort of individuals with type 2 diabetes, higher levels of circulating SMs were associated with increased risk of CVD [30]. In populations without diabetes, evidence has been inconsistent on the associations of SM and CVD [31–33]. In a previous study including a large type 1 diabetes population, higher levels of SMs were associated with an increased risk of coronary heart disease in a model adjusted for sex, age, diabetes duration and smoking, but not after further adjustment [34]. Our findings implicate novel avenues for understanding the relationship between type 1 diabetes and CVD, that need to be explored.

One of the limitations of this study is that metabolites and molecular lipid species might be modulated by diet and exercise, which we were unable to adjust for [35–38]. The absence of information on changes in medication is another limitation, particularly regarding changes in cholesterol lowering drugs. Furthermore, the low number of specific CVE is a limiting factor, as well as the lack of a replication cohort. On the other hand, the present study includes a large, well-defined, type 1 diabetes population with up to seven years of longitudinal follow up from validated registers. Additionally, the comprehensive metabolomic and lipidomic analyses are a main strength of this study.

## Conclusions

To summarize, we showed an association between the circulating metabolites 4-HPAA and threonine and risk of future cardiovascular events. Furthermore, we demonstrated that several molecular lipid species previously associated with microvascular complications, were also found to be associated with macrovascular complications. This suggests a potential overlap in the molecular pathophysiology of micro- and macrovascular complications, independent of traditional risk factors. While the causal effect of these biomolecules on the cardiovascular system remains unknown, our findings support that omics-based technologies, although still in an early phase, may have the potential to unravel new pathways and biomarkers in the field of cardiovascular disease in type 1 diabetes.

## Supplementary Information

**Additional file 1: Table S1.** Diagnoses and codes included in the CVE endpoints. **Table S2.** List of analyzed metabolites. **Table S3.** List of analyzed molecular lipid species. **Figure S1.** Correlation between metabolites and clinical lipid measurements and statin treatment. **Figure S2.** Correlation between molecular lipid species and clinical lipid measurements and statin treatment.

## Data Availability

The datasets generated and/or analyzed during the current study are not publicly available due to Danish GDPR law but are available from the corresponding author on reasonable request.

## Abbreviations

4-HPAA: 4-Hydroxyphenylacetic acid
α-KG: Alpha-ketoglutaric acid
BMI: Body mass index
CI: Confidence interval
CV: Cardiovascular
CVD: Cardiovascular disease
CVE: Cardiovascular events
eGFR: Estimated glomerular filtration rate
FDR: False discovery rate
HbA1C: Glycated hemoglobin
HR: Hazard ratios
LDL: Low-density lipoprotein
LPC: Lyso-phosphatidylcholines
PC: Diacyl-phosphatidylcholines
PC-O: Alkyl-acyl-phosphatidylcholines
SM: Sphingomyelin
TCA: Tricarboxylic acid
TG: Triacylglycerols
UAER: Urinary albumin excretion rate
