# Supplementary material for: Circulating metabolites and molecular lipid species are associated with future cardiovascular morbidity and mortality in type 1 diabetes
Source: Cardiovasc Diabetol. 2022 Jul 18;21:135. doi: 10.1186/s12933-022-01568-8 (PMC9295441; doi:10.1186/s12933-022-01568-8)
Supplement: Supplementary file 1 — Additional file 1: Table S1. Diagnoses and codes included in the CVE endpoints. Table S2. List of analyzed metabolites. Table S3. List of analyzed molecular lipid species. Figure S1. Correlation between metabolites and clinical lipid measurements and statin treatment. Figure S2. Correlation between molecular lipid species and clinical lipid measurements and statin treatment. [file 12933_2022_1568_MOESM1_ESM.docx]

**Table S1**

|  | **Diagnosis or procedure** | **Classifier** | **Codes** |
| --- | --- | --- | --- |
| CVE | Myocardial infarction | ICD-10 | I21-I24 |
|  | Non-fatal stroke | ICD-10 | I61-I66 |
|  | Coronary arterial intervention | NCSP | KFNA-KFND |
|  | Peripheral arterial intervention | NCSP | KP: A-F  – E, F, H, Q |
|  |  |  |  |

Table S1. Diagnoses and codes included in the CVE endpoints.

CVE: Cardiovascular event.

ICD-10: International Statistical Classification of Diseases and Related Health Problems 10^th^ Ed.

NCSP: Nordic Medico-Statistical Committee Classification of Surgical Procedures

**Table S2**

*List of analyzed metabolites.*

| Name |
| --- |
| 1-Dodecanol |
| 1-Monopalmitin |
| 1,3-Propanediol |
| 11-Eicosenoic acid |
| 2-hydroxy Isovaleric acid |
| 2-Hydroxybutyric acid |
| 2-Palmitoylglycerol |
| 2,4-Dihydroxybutanoic acid |
| 3-Hydroxybutyric acid |
| 3-Indoleacetic acid |
| 3-Indolepropionic acid |
| 3,4-Dihydroxybutanoic acid |
| 4-Deoxytetronic acid (1) |
| 4-Deoxytetronic acid (2) |
| 4-Hydroxybenzeneacetic acid |
| 4-Hydroxybutanoic acid |
| 4-Hydroxyphenyllactic acid |
| Alanine |
| alpha-ketoglutaric acid |
| alpha-Tocopherol |
| Aminomalonic acid |
| Arabinopyranose |
| Arachidic acid |
| Arachidonic acid |
| Benzeneacetic acid |
| Bisphenol A |
| Campesterol |
| Cholesterol |
| Citric acid |
| Creatinine |
| Decanoic acid |
| Docosahexaenoic acid |
| Dodecanoic acid |
| Eicosapentaenoic acid |
| Ethanolamine |
| Fumaric acid |
| Glutamic acid |
| Glyceric acid |
| Glycerol (1) |
| Glycerol (2) |
| Glyceryl-glycoside |
| Glycine |
| Heptadecanoic acid (1) |
| Heptadecanoic acid (2) |
| Hydroxylamine |
| Hydroxyproline |
| Isoleucine |
| L-5-Oxoproline |
| Lactic acid |
| Leucine |
| Linoleic acid |
| Malic acid |
| Methionine |
| Myo inositol |
| Myristoleic acid |
| Nonadecanoic acid |
| Nonanoic acid |
| Octanoic acid |
| Oleic acid |
| Palmitic acid |
| Phenylalanine |
| Proline |
| Pyroglutamic acid |
| Pyruvic acid |
| Ribitol (1) |
| Ribitol (2) |
| Ribonic acid |
| Serine |
| Stearic acid |
| Succinic acid |
| Tartronic acid |
| Threonine |
| Tridecanoic acid |
| Tyrosine |
| Valine |

**Table S3**

*List of analyzed molecular lipid species.*

| Name |
| --- |
| LPC(16:0) |
| LPC(16:1) |
| LPC(18:0) |
| LPC(18:1) |
| LPC(18:2) |
| LPC(20:4) |
| PC(16:0e/18:1(9Z)) |
| PC(32:0) |
| PC(32:1) |
| PC(32:2) |
| PC(33:1) |
| PC(34:1) |
| PC(34:2) |
| PC(34:3) |
| PC(35:1) |
| PC(35:2) |
| PC(36:2) |
| PC(36:3) |
| PC(36:4) |
| PC(36:5) |
| PC(37:2) |
| PC(38:2) |
| PC(38:3) |
| PC(38:4) |
| PC(38:5) |
| PC(38:6) |
| PC(40:5) |
| PC(40:6) |
| PC(40:7) |
| PC(O-34:2) |
| PC(O-34:3) |
| PC(O-36:2) |
| PC(O-36:3) |
| PC(O-36:4) |
| PC(O-36:5) |
| PC(O-38:4) |
| PC(O-38:5) |
| PC(O-38:6) |
| SM(42:2) |
| SM(d16:1/18:1) or SM(d18:2/16:0) |
| SM(d18:1/24:0) |
| SM(d18:1/24:0) or SM(d18:0/24:1) |
| SM(d18:2/24:1) |
| SM(d32:1) |
| SM(d33:1) |
| SM(d34:1) |
| SM(d36:1) |
| SM(d36:2) |
| SM(d38:1) |
| SM(d38:2) |
| SM(d39:1) |
| SM(d40:1) |
| SM(d40:2) |
| SM(d41:1) |
| SM(d41:2) |
| TG(14:0/16:0/18:1) |
| TG(14:0/18:1/18:1) |
| TG(14:0/18:2/18:2) |
| TG(16:0/18:0/18:1) |
| TG(16:0/18:2/18:2) |
| TG(16:0/18:2/18:3) |
| TG(16:0/18:2/22:6) |
| TG(16:0/22:5/18:1) or TG(20:4/18:1/18:1) |
| TG(18:0/18:1/20:4) |
| TG(18:1/12:0/18:1) or TG(18:2/16:0/14:0) |
| TG(18:1/18:1/16:0) |
| TG(18:1/18:1/18:1) |
| TG(18:1/18:1/22:6) |
| TG(18:1/18:2/18:2) |
| TG(18:2/18:1/16:0) |
| TG(18:2/18:1/18:1) |
| TG(18:2/18:2/18:2) or TG(18:3/18:2/18:1) |
| TG(18:2/22:5/16:0) |
| TG(45:0) |
| TG(46:0) |
| TG(46:1) |
| TG(46:2) |
| TG(47:1) |
| TG(48:3) |
| TG(49:1) |
| TG(49:2) |
| TG(49:3) |
| TG(50:0) |
| TG(50:1) |
| TG(50:2) |
| TG(50:3) |
| TG(51:1) |
| TG(51:2) |
| TG(51:3) |
| TG(52:2) |
| TG(52:3) |
| TG(52:4) |
| TG(52:5) |
| TG(53:2) |
| TG(53:3) |
| TG(53:4) |
| TG(54:2) |
| TG(54:3) |
| TG(54:4) |
| TG(54:5) |
| TG(54:6) |
| TG(56:3) |
| TG(56:4) |
| TG(56:5) |
| TG(56:6) |
| TG(56:7) |
| TG(58:9) |


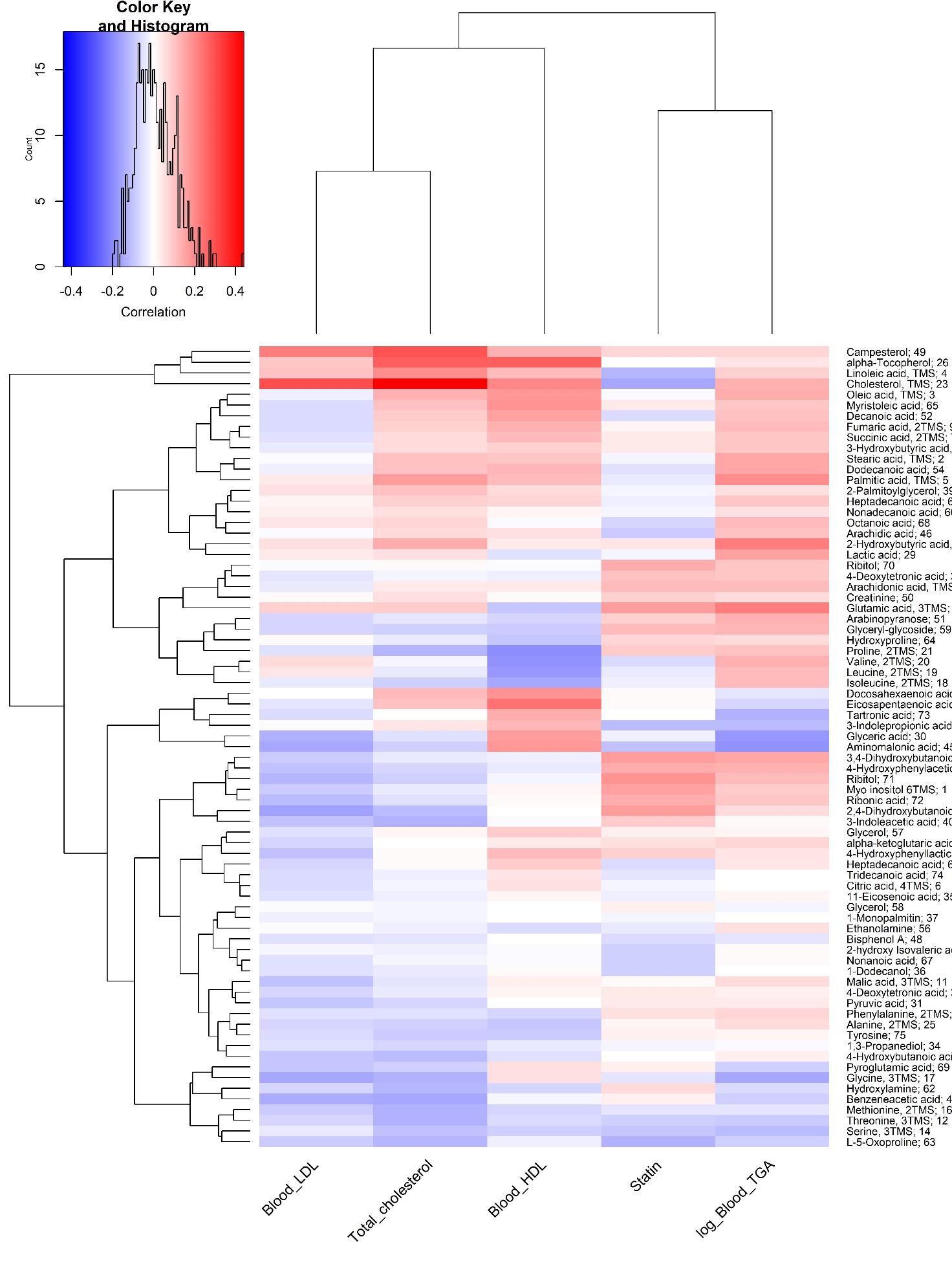


Figure S1: Correlation between metabolites and clinical lipid measurements and statin treatment.


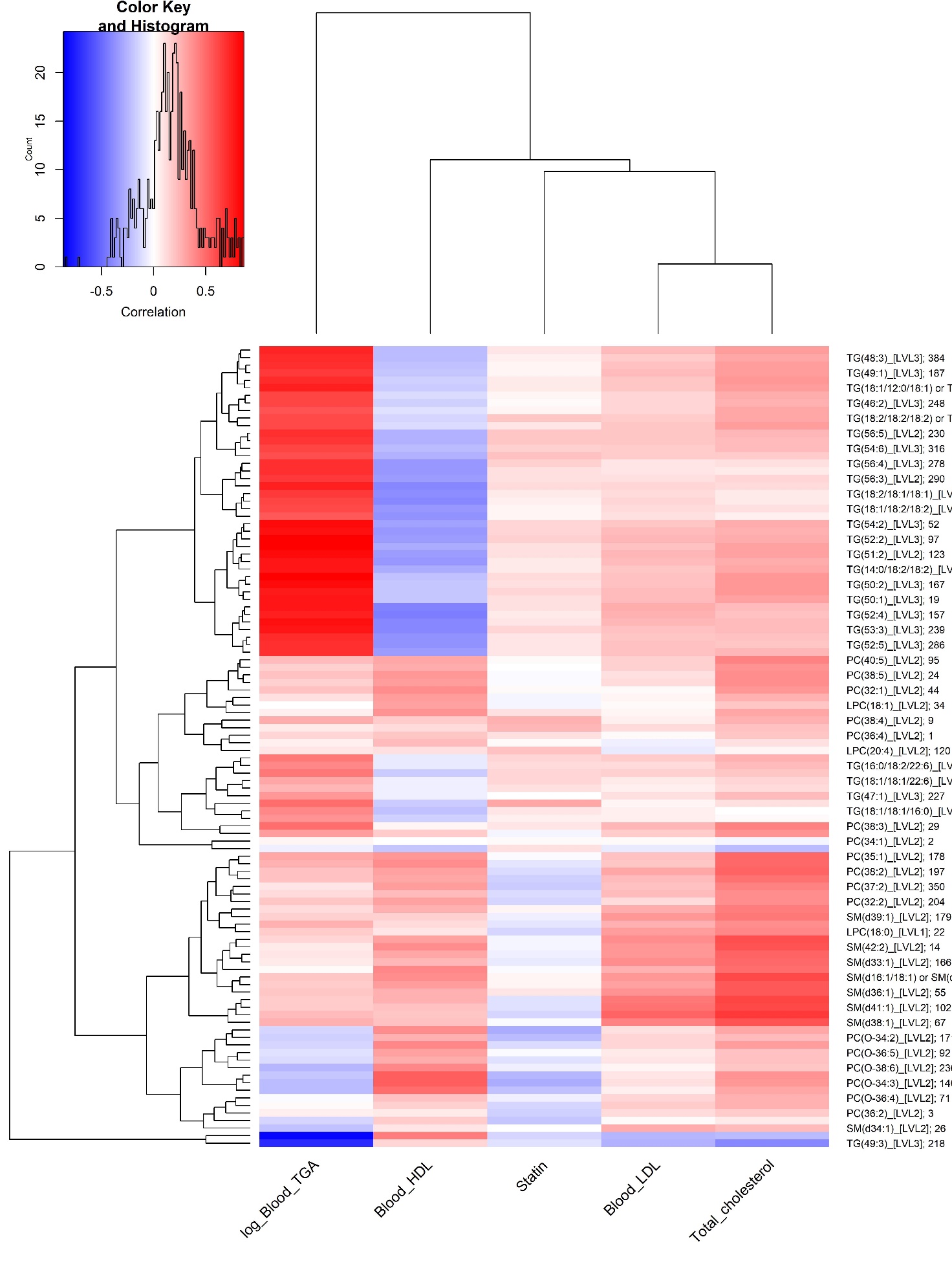


Figure S2: Correlation between molecular lipid species and clinical lipid measurements and statin treatment. PC: Phosphatidylcholine, PC-O: alkyl-acyl-phosphatidylcholine, LPC: Lyso-phosphatidylcholine, TG: Triglyceride, SM: Sphingomyelin.
